# A Noise Removal Method for Uniform Circular Arrays in Complex Underwater Noise Environments with Low SNR

**DOI:** 10.3390/s17061345

**Published:** 2017-06-09

**Authors:** Huijun Xia, Kunde Yang, Yuanliang Ma, Yong Wang, Yaxiong Liu

**Affiliations:** 1 School of Marine Science and Technology, Northwestern Polytechnical University, Xi’an 710072, China; xiahuijun1122@mail.nwpu.edu.cn (H.X.); ylma@nwpu.edu.cn (Y.M.); swadqe09@mail.nwpu.edu.cn (Y.L.); 2 Key Laboratory of Ocean Acoustics and Sensing, Ministry of Industry and Information Technology, Xi’an 710072, China; 3 School of Mechanical Engineering, Xi’an Jiaotong University, Xi’an 710072, China; yongwang.nwpu@gmail.com

**Keywords:** underwater ambient noise, sensor array signal processing, signal-to-noise ratio, beamforming, noise removal

## Abstract

Generally, many beamforming methods are derived under the assumption of white noise. In practice, the actual underwater ambient noise is complex. As a result, the noise removal capacity of the beamforming method may be deteriorated considerably. Furthermore, in underwater environment with extremely low signal-to-noise ratio (SNR), the performances of the beamforming method may be deteriorated. To tackle these problems, a noise removal method for uniform circular array (UCA) is proposed to remove the received noise and improve the SNR in complex noise environments with low SNR. First, the symmetrical noise sources are defined and the spatial correlation of the symmetrical noise sources is calculated. Then, based on the preceding results, the noise covariance matrix is decomposed into symmetrical and asymmetrical components. Analysis indicates that the symmetrical component only affect the real part of the noise covariance matrix. Consequently, the delay-and-sum (DAS) beamforming is performed by using the imaginary part of the covariance matrix to remove the symmetrical component. However, the noise removal method causes two problems. First, the proposed method produces a false target. Second, the proposed method would seriously suppress the signal when it is located in some directions. To solve the first problem, two methods to reconstruct the signal covariance matrix are presented: based on the estimation of signal variance and based on the constrained optimization algorithm. To solve the second problem, we can design the array configuration and select the suitable working frequency. Theoretical analysis and experimental results are included to demonstrate that the proposed methods are particularly effective in complex noise environments with low SNR. The proposed method can be extended to any array.

## 1. Introduction

Array signal processing is extensively used for noise removal and signal enhancement in underwater environments with low SNR (see, e.g., [1,2,3]). The delay-and-sum (DAS) beamforming and the minimum variance distortionless response (MVDR) [4] methods are the two most important beamforming methods. The MVDR method is a well-known data-dependent filter which is aimed at minimizing the energy of noise and interference coming from different directions, while keeping a fixed gain on the desired direction of arrival. The robustness of the MVDR method is related to the number of snapshots, channel amplitude and phase errors, input SNR, and position errors. However, the MVDR method is not robust in many practical applications. A popular approach to improve the robustness is the diagonal loading method presented in [5]. The main limitation of the diagonal loading method is that the process of efficiently picking the penalty weight is not clear, although useful data-dependent methods have been proposed for specific applications (see, e.g., [6,7]). By contrast, the DAS method is more robust, so it is widely used in the underwater array signal processing. However, the noise removal capacity of the method is limited by the array aperture. As a consequence, the low SNR environment seriously degrades the performance of this method. 

Another problem is that the actual underwater ambient noise is complex (see, e.g., [8,9,10,11]), and the noise spatial correlation function [9] is not equal to zero. The received noises of the two arbitrary array elements are correlated. Typically, the beamforming methods are derived under the assumption of white noise, whose noise covariance matrix is a scaled identity matrix. Although this assumption has been observed to be valid in many applications, it may be occasionally violated and yield poor performance because, in practice, the noise spatial correlation function [9] is not equal to zero. 

In conclusion, studying the array signal processing method in complex noise environments with low SNR is a challenging task. To address this problem, a large number of strategies have been proposed. On the one hand, the noise is removed by estimating the noise parameters. In [12], the noise field is assumed as a colored noise field, and the direction of arrival and noise parameters are estimated by maximum- likelihood estimator. In [13,14], the noise covariance matrix is assumed to keep a diagonal structure, but the diagonal entries are not identical to each other. Then, the noise subspace is obtained. On the other hand, the noise is removed by eliminating the real part of the covariance matrix. A research group proposed a high-resolution bearing estimation method for weak signals in non-white noise field in [15] and compared the performance of the multiple-signal classification (MUSIC) method with that of the MUSIC method in which only the imaginary part of the covariance matrix is applied in [16]. These methods provide better bearing estimation and high resolution in a low SNR environment under an ideal noise model. In practice, the ideal model cannot be entirely satisfied, and the noise is always complex in underwater environments. Thus, these methods are limited. To address this problem, the authors proposed a new noise reduction method that is suitable for complex noise environments with low SNR (see, e.g., [17,18]). Another serious problem is that the aforementioned methods may seriously suppress the signal (see, e.g., [15,16]); thus, the signal cannot be detected. To address this problem, we proposed two methods in [17], but the use of these two methods is limited. We also designed the array configuration and selected the suitable working frequency in [18]. In [17,18], the noise reduction method is based on the assumption that the noise sources are located in a plane, which is not practical. By contrast, the noise reduction method proposed in this manuscript is based on the fact that the actual received noise is from three- dimensional space. Consequently, the proposed method is more generally applicable and can be extended to any array.

In this study, a noise removal method is proposed. The received noise (see, e.g., [19,20,21,22,23,24]), which is from the 3D space, can be accurately modeled by adding the fields from a large number of uncorrelated sources (see, e.g., [24,25,26,27]). Theoretically, the noise covariance matrix is decomposed into symmetrical and asymmetrical components. Therefore, the imaginary part of the covariance matrix is used in the DAS method to remove symmetrical noise in which a false target appears. To address the problem, a method of reconstructing the signal covariance matrix is presented to eliminate the false target based on the constrained optimization method (CP-RCMDAS) presented in [17,18]. In this study, two methods of reconstructing the signal covariance matrix are presented to eliminate the false target: based on the signal variance estimation method (SVE-RCMDAS) and CP-RCMDAS. The advantages and disadvantages of the two methods are first compared. Theoretical analysis and experimental results show that the proposed method, which is easy to implement, improves the noise removal capacity and the output SNR of the DAS method in complex underwater environments with low SNR.

The remainder of this paper is organized as follows: Section 2 describes the signal model, gives the definition of the symmetrical noise sources, and the spatial correlation of these two symmetrical noise sources is derived. Section 3 gives the principle of the noise covariance matrix decomposition. The noise removal method and its performance are provided in Section 4. Both simulation results provided in Section 5 and experimental results provided in Section 6 demonstrate the validity of our proposals. Finally, Section 7 concludes this paper.

## 2. Background

In this section, the received signal model of the array is established, and then the definition of the symmetrical noise sources is presented. Finally, the spatial correlation of the two symmetrical noise sources is derived, which is the basis of the noise covariance matrix decomposition in the next section.

### 2.1. Array Model

**Figure 1:**
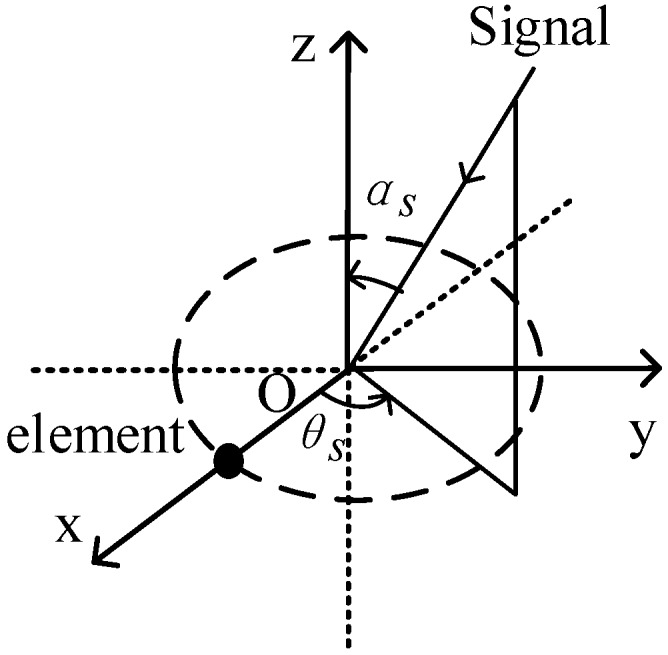
Array configuration.

An *M*-element UCA, which is located on the XY plane of a Cartesian coordinate system, is shown in Figure 1. The element on the *ox* axis is labeled as 1, the other elements are labeled as 2, 3, …, and *M* in the counterclockwise direction. The radius is r. The angle subtended between two adjacent elements is denoted as β=2π/M. Consequently, the angle of the *m*th element is $\beta_{m}=(m-1)\beta$.

The position vector of the array element is described as:

(1) $$P_{m}={\left[r\cos\beta_{m},r\sin\beta_{m},0\right]}^{T},m=1,2,\cdots ,M.$$

The UCA captures a zero-mean source signal whose center frequency is f, variance is $\sigma_{s}^{2}$, azimuth angle is $\theta_{s}\in [-\pi ,\pi )$, and elevation angle is $\alpha_{s}\in [0,\pi ]$. The signal is uncorrelated to the noise. The unitary vector indicating the signal direction of arrival can be described as:

(2) $$v_{s}(\alpha_{s},\theta_{s})=-{\left[\sin\alpha_{s}\cos\theta_{s},\sin\alpha_{s}\sin\theta_{s},\cos\alpha_{s}\right]}^{T}.$$

The signal waveform is denoted as s(t). Thus, the received signal and noise of the *m*th element are easily obtained as:

(3) $$x_{m}(t)=s(t)e^{-jk_{s}^{T}(\alpha_{s},\theta_{s})P_{m}}+n_{m}(t),$$

where $n_{m}(t)$ is the received noise, which is assumed to be a zero-mean random process; $k_{s}(\alpha_{s},\theta_{s})$ is the wave number, which can be calculated by using $k_{s}(\alpha_{s},\theta_{s})=\omega v_{s}(\alpha_{s},\theta_{s})/c$; w=2πf is the angular frequency; and c is the sound velocity. The received signal and noise of UCA are acquired as:

(4) $$X(t)=\left[\begin{array}{c} e^{-jk_{s}^{T}P_{1}}\\ e^{-jk_{s}^{T}P_{2}}\\ \vdots \\ e^{-jk_{s}^{T}P_{M}} \end{array}\right]s(t)+\left[\begin{array}{c} n_{1}(t)\\ n_{2}(t)\\ \vdots \\ n_{M}(t) \end{array}\right]={\tilde{a}}_{s}(\alpha_{s},\theta_{s})s(t)+N(t),$$

where $X(t)={\left[\begin{array}{cccc} x_{1}(t) & x_{2}(t) & \cdots & x_{M}(t) \end{array}\right]}^{T}$, ${\tilde{a}}_{s}(\alpha_{s},\theta_{s})$ is the response vector of the signal, and N(t) is the received noise vector. Then, the covariance matrix of the received data is calculated by:

(5) $$R=E[XX^{H}]=R_{s}+R_{n},$$

where $R_{s}$ is the signal covariance matrix and $R_{n}$ is the noise covariance matrix. $R_{s}$ is denoted as:

(6) $$R_{s}=\left[\begin{array}{cccc} \sigma_{s}^{2} & \sigma_{s}^{2}e^{j\Delta_{12}(\alpha_{s},\theta_{s})} & \cdots & \sigma_{s}^{2}e^{j\Delta_{1M}(\alpha_{s},\theta_{s})}\\ \sigma_{s}^{2}e^{j\Delta_{21}(\alpha_{s},\theta_{s})} & \sigma_{s}^{2} & \cdots & \sigma_{s}^{2}e^{j\Delta_{2M}(\alpha_{s},\theta_{s})}\\ \vdots & \vdots & \ddots & \vdots \\ \sigma_{s}^{2}e^{j\Delta_{M1}(\alpha_{s},\theta_{s})} & \sigma_{s}^{2}e^{j\Delta_{M2}(\alpha_{s},\theta_{s})} & \cdots & \sigma_{s}^{2} \end{array}\right],$$

where $\Delta_{kl}(\alpha_{s},\theta_{s})=\sin\alpha_{s}\left[\cos(\beta_{k}-\theta_{s})-\cos(\beta_{l}-\theta_{s})\right]\omega r/c$ is defined as the phase difference. For notational simplicity, Equation (6) is denoted by $R_{s}={\left(\sigma_{s}^{2}e^{j\Delta_{kl}(\alpha_{s},\theta_{s})}\right)}_{M\times M}$, where k denotes the row number and l denotes the column number. The similar matrices, which will be presented later, are denoted by the same style.

### 2.2. Symmetrical Noise Sources

The definition of the symmetrical noise sources is presented. Two received sensors are denoted as A and B. Moreover, two noise sources exist. The received narrowband noise signals radiated by one noise source are denoted as $N_{A1}(t)$ and $N_{B1}(t)$, respectively, which satisfy:

(7) $$N_{B1}(t)=N_{A1}(t)e^{-j\Delta_{1}},$$

where $\Delta_{1}$ is the phase difference. The received narrowband noise signals radiated by another noise source are denoted as $N_{A2}(t)$ and $N_{B2}(t)$, respectively, which are independent of $N_{A1}(t)$ and $N_{B1}(t)$. As a result, we can obtain:

(8) $$N_{B2}(t)=N_{A2}(t)e^{-j\Delta_{2}},$$

where $\Delta_{2}$ is the phase difference. If the noise variances of the two received noise signals are equal and the phase differences are opposite, that is:

(9) $$\begin{array}{l} E[N_{A1}^{2}(t)]=E[N_{A2}^{2}(t)]=\sigma_{A}^{2}\\ \Delta_{1}=-\Delta_{2} \end{array},$$

then the two noise sources are defined as symmetrical noise sources, and the noise field generated by them is denoted as a symmetrical noise field.

### 2.3. Spatial Correlation of Symmetrical Noise Sources

According to Section 2.2, the received noise signal of sensor A is described as:

(10) $$N_{A}(t)=N_{A1}(t)+N_{A2}(t).$$

According to Equations (7)–(9), the received noise signal of sensor B is described as:

(11) $$N_{B}(t)=N_{B1}(t)+N_{B2}(t)=N_{A1}(t)e^{-j\Delta_{1}}+N_{A2}(t)e^{j\Delta_{1}}.$$

The correlation function can be obtained as:

(12) $$r_{AB}=E[N_{A}(t)N_{B}^{*}(t)]=E[N_{A1}^{2}(t)]e^{j\Delta_{1}}+E[N_{A2}^{2}(t)]e^{-j\Delta_{1}}=2\sigma_{A}^{2}\cos\Delta_{1}.$$

Equation (12) shows that the symmetrical noise sources only affect the real part of the correlation function, and the imaginary part is zero. This result will be the basis of the noise covariance matrix decomposition in the next section.

## 3. Noise Covariance Matrix Decomposition

In this Section, the principle of noise covariance matrix decomposition is presented. Based on the Section 2.2 and Section 2.3, the symmetrical noise sources cannot affect the imaginary part of the correlation function. In practice, the received noise signal of the sensor array is from 3D space, not from two symmetrical noise sources. Thus, the decomposition of the noise covariance matrix should be studied in this section under the actual underwater ambient noise.

The received noise can be accurately modeled by adding the fields from a large number of uncorrelated sources (see, e.g., [24,25,26,27]). The noise field is assumed to be generated by the *G* noise sources, which are denoted as $N_{g}$, *g* = 1, 2, …, *G*. The center frequency is *f*, the azimuth angle is $\varphi_{g}\in [-\pi ,\pi )$, the elevation angle is $\alpha_{g}\in [0,\pi ]$, and the noise variance is $\sigma_{g}^{2}$. Thus, the received noise of the *m*th element is obtained:

(13) $$n_{m}(t)=\sum_{g=1}^{G}N_{g}(t)e^{j\omega \;r\sin\alpha_{g}\cos(\beta_{m}-\varphi_{g})/c},$$

where the received noise from $N_{g}$ is represented as $N_{g}(t)$. Then, the noise covariance matrix in Equation (5) is obtained as:

(14) $$R_{n}={\left(\sum_{g=1}^{G}\sigma_{g}^{2}e^{j\Delta_{kl}(\alpha_{g},\varphi_{g})}\right)}_{M\times M}.$$

where:

(15) $$\Delta_{kl}(\alpha_{g},\varphi_{g})=\sin\alpha_{g}\left[\cos(\beta_{k}-\varphi_{g})-\cos(\beta_{l}-\varphi_{g})\right]\omega r/c$$

is defined as the phase difference.

We take one off-diagonal element of noise covariance matrix in Equation (14) to discuss:

(16) $$r_{kl}=\sum_{g=1}^{G}\sigma_{g}^{2}e^{j\Delta_{kl}(\alpha_{g},\varphi_{g})},$$

where k≠l. The phase difference can be simplified into:

(17) $$\Delta_{kl}(\alpha_{g},\varphi_{g})=\sqrt{c_{1}^{2}+c_{2}^{2}}\sin\alpha_{g}\sin(\varphi_{g}+\upsilon_{kl})\omega r/c,$$

where $c_{1}=\cos\beta_{k}-\cos\beta_{l}$, $c_{2}=\sin\beta_{k}-\sin\beta_{l}$, and $\upsilon_{kl}=\arg\tan(c_{1}/c_{2})$. For all noise sources, we can obtain $\alpha_{g}\in [0,\pi ]$ and $\varphi_{g}\in [-\pi ,\pi )$. Thus, the partial derivatives of $\Delta_{kl}(\alpha_{g},\varphi_{g})$ with respect to $\alpha_{g}$ and $\varphi_{g}$ are, respectively, given by:

(18) $$\begin{array}{l} \frac{\partial \Delta_{kl}(\alpha_{g},\varphi_{g})}{\partial \alpha_{g}}=c_{3}\cos\alpha_{g}\sin(\varphi_{g}+\upsilon_{kl})\\ \frac{\partial \Delta_{kl}(\alpha_{g},\varphi_{g})}{\partial \varphi_{g}}=c_{3}\sin\alpha_{g}\cos(\varphi_{g}+\upsilon_{kl}) \end{array},$$

where $c_{3}=\omega r/c\sqrt{c_{1}^{2}+c_{2}^{2}}$. Therefore, setting the derivative in Equation (18) as zero, we obtain two sets of $\alpha_{g}$ and $\varphi_{g}$, which correspond to the two extremes of the phase difference:

(19) $$\left\{ \begin{array}{l} \alpha_{g}=0,\pi \\ \varphi_{g}=k\pi -\upsilon_{kl} \end{array}\right.\left\{ \begin{array}{l} \alpha_{g}=\frac{\pi }{2}\\ \varphi_{g}=\frac{k\pi }{2}-\upsilon_{kl} \end{array}\right.,$$

where $\varphi_{g}$ satisfies $\varphi_{g}\in [-\pi ,\pi )$ by selecting a suitable *k*.

When $\alpha_{g}=\pi /2$, two values of $\varphi_{g}$ cause the phase difference to be the maximum and minimum. Moreover, according to Equation (17), we can obtain:

(20) $$\max\left[\Delta_{kl}(\alpha_{g},\varphi_{g})\right]=c_{3},\;\min\left[\Delta_{kl}(\alpha_{g},\varphi_{g})\right]=-c_{3}.$$

According to Equation (20), the maximum is the negative of the minimum, and the phase difference is continuous when $\alpha_{g}\in [0,\pi ]$ and $\varphi_{g}\in [-\pi ,\pi )$. Consequently, two noise sources, namely, $N_{1}$ and $N_{2}$, whose variances are denoted as $\sigma_{1}^{2}$ and $\sigma_{2}^{2}$, must be existed, and the phase differences satisfy the equation $\Delta_{kl}(\alpha_{1},\varphi_{1})=-\Delta_{kl}(\alpha_{2},\varphi_{2})$.

Then, the noise field generated by $N_{1}$ and $N_{2}$ can be decomposed into symmetrical and asymmetrical noise fields. The decomposition method is as follows: If $\sigma_{1}^{2}>\sigma_{2}^{2}$, then the noise source $N_{1}$ is decomposed into two noise sources, namely, ${N^{\prime }}_{1}$ and $N_{1\Delta }$, and the corresponding variances are equal to $\sigma_{2}^{2}$ and $\sigma_{p}$, respectively. The noise sources ${N^{\prime }}_{1}$ and $N_{2}$ conform to Equation (9), so they generate the symmetrical noise field, whereas $N_{1\Delta }$ generates the asymmetrical noise field. $N_{1}$ and $N_{2}$ are arbitrary, so we can infer that the noise field generated by all *G* noise sources can be decomposed. The basic idea of the decomposition is that all noise sources that have equal phase differences, are equivalent to a noise source. Then, some equivalent noise sources are obtained. Finally, the noise field generated by these equivalent noise sources is decomposed into symmetrical and asymmetrical noise fields. The specific process is as follows:

(1) All noise sources can be divided into B sets, which are denoted as $G_{b},b=1,2,\cdots ,B$. The principle of dividing the set is that the phase differences of all noise sources in the set $G_{b}$ are equal, denoted as ${\left(\Delta_{kl}\right)}_{b}$. Therefore, the noise sources in the set of $G_{b}$ are equivalent to a noise source denoted as ${\tilde{N}}_{b}$. Consequently, *B* equivalent noise sources are obtained.
(2) The phase differences of ${\tilde{N}}_{b},b=1,2,\cdots ,B$ conform to Equation (20). Consequently, for arbitrary ${\tilde{N}}_{b}$, another noise source must be existed, without losing generality, denoted as ${\tilde{N}}_{B-b+1}$, whose phase difference satisfies ${\left(\Delta_{kl}\right)}_{B-b+1}=-{\left(\Delta_{kl}\right)}_{b}$. Consequently, the noise sources of $G_{b}$ and $G_{B-b+1}$ can be decomposed. Thus, the summation is calculated by:
  (21) $$\varepsilon_{b}=\sum_{g\in G_{b}}\sigma_{g}^{2}e^{j\Delta_{kl}(\alpha_{g},\varphi_{g})}+\sum_{g\in G_{B-b+1}}\sigma_{g}^{2}e^{j\Delta_{kl}(\alpha_{g},\varphi_{g})}.$$
  $\varepsilon_{b}$ is simplified into:
  (22) $$\varepsilon_{b}=E_{1}e^{j{\left(\Delta_{kl}\right)}_{b}}+E_{2}e^{-j{\left(\Delta_{kl}\right)}_{b}}={P^{\prime }}_{b}\cos({\left(\Delta_{kl}\right)}_{b})+P_{b}e^{j\mu_{b}{\left(\Delta_{kl}\right)}_{b}},$$
  where ${P^{\prime }}_{b}=2\min(E_{1},E_{2})$, $P_{b}=\max(E_{1},E_{2})-\min(E_{1},E_{2})$, and $E_{1}$, $E_{2}$ are denoted as $E_{1}=\sum_{g\in G_{b}}\sigma_{g}^{2}$ and $E_{2}=\sum_{g\in G_{B-b+1}}\sigma_{g}^{2}$, respectively. If $E_{1}>E_{2}$, then $\mu_{b}=1$; otherwise, $\mu_{b}=-1$.
(3) For all *G* noise sources, the off-diagonal element of the noise covariance matrix in Equation (16) is simplified into:
  (23) $$r_{kl}=\sum_{b=1}^{B^{\prime }}\varepsilon_{b}=\sum_{b=1}^{B^{\prime }}\left({P^{\prime }}_{klb}\cos({\left(\Delta_{kl}\right)}_{b})+P_{klb}e^{j\mu_{klb}{\left(\Delta_{kl}\right)}_{b}}\right),$$
  where $B^{\prime }=B/2$. Different *k* and *l* result in different $G_{b},b=1,2,\cdots ,B$. Thus, ${P^{\prime }}_{klb}$, $\;P_{klb}$, and $\mu_{klb}$ change with *k* and *l*.
(4) All off-diagonal elements of the noise covariance matrix in Equation (14) can be analyzed by the same procedure.

Finally, Equation (23) shows that the symmetrical component affects the real part but not the imaginary part of the noise covariance matrix. Therefore, we can infer that most of the noise can be removed by using only the imaginary part of the covariance matrix in the array signal processing to improve the output SNR.

## 4. Noise Removal Method

According to Section 2, the noise removal method can improve the output SNR and noise removal capacity. In this section, we take the DAS method as an example to present the imaginary DAS (IDAS) beamforming method in Section 4.1 and the performance of the IDAS method is presented in Section 4.2. The result shows that the IDAS method generates a false target. Moreover, Section 4.3 provides two methods to address this problem.

### 4.1. IDAS Method

This paper takes the DAS method as an example. The output variance of the DAS method is described as:

(24) $$\begin{array}{ll} P_{DAS}(\alpha ,\theta ) & =w^{H}(\alpha ,\theta )Rw(\alpha ,\theta )\\ & =w^{H}(\alpha ,\theta )R_{s}w(\alpha ,\theta )+w^{H}(\alpha ,\theta )R_{n}w(\alpha ,\theta )\\ & =P_{so}+P_{no} \end{array},$$

where w(α,θ)=a(α,θ)/M is the array weight vector, a(α,θ) is the array manifold vector, θ and α are the scanning azimuth angle and scanning elevation angle, respectively. Moreover, $P_{so}$ and $P_{no}$ denote the output signal and output noise variances, respectively. The output SNR can be obtained as:

(25) $$SNR_{o}=\frac{P_{so}}{P_{no}}=\frac{\sigma_{s}^{2}}{\left(\sum_{g=1}^{G}\sigma_{g}^{2})w^{H}(\alpha_{s},\theta_{s})\rho_{n}w(\alpha_{s},\theta_{s}\right)},$$

where $\rho_{n}$ is the normalized noise covariance matrix.

The IDAS method is implemented by using only the imaginary part of the covariance matrix, which is used in the DAS method. Thus, the output intensity is obtained as:

(26) $$P_{IDAS}(\alpha ,\theta )=w^{H}(\alpha ,\theta )R_{im}w(\alpha ,\theta ),$$

where $R_{im}$ is the imaginary part of R.

### 4.2. Performance of the IDAS Method

The performance of the IDAS method is analyzed in this subsection. Firstly, the signal contained in the real part is also lost, so the signal loss is analyzed. According to Equation (6), the imaginary part of the signal covariance matrix is obtained as:

(27) $$R_{is}={\left(j\sigma_{s}^{2}\sin(\Delta_{kl}(\alpha_{s},\theta_{s}))\right)}_{M\times M},$$

where *j* is the imaginary unit.

The off-diagonal element of $R_{is}$ is simplified into:

(28) $$j\sigma_{s}^{2}\sin(\Delta_{kl}(\alpha_{s},\theta_{s}))\;=\frac{\sigma_{s}^{2}}{2}e^{j\Delta_{kl}(\alpha_{s},\theta_{s})}-\frac{\sigma_{s}^{2}}{2}e^{-j\Delta_{kl}(\alpha_{s},\theta_{s})}.$$

According to Equation (28), $R_{is}$ can be described as the difference between the matrices $R_{is1}$ and $R_{is2}$, which are expressed as:

(29) $$R_{is1}={\left(\frac{\sigma_{s}^{2}}{2}e^{j\Delta_{kl}(\alpha_{s},\theta_{s})}\right)}_{M\times M},\;R_{is2}={\left(\frac{\sigma_{s}^{2}}{2}e^{-j\Delta_{kl}(\alpha_{s},\theta_{s})}\right)}_{M\times M}.$$

$R_{is1}$ and $R_{is2}$ can be viewed as two signal covariance matrices: one is the actual target whose azimuth angle, elevation angle, and variance are $\theta_{s}$, $\alpha_{s}$, and $\sigma_{s}^{2}/2$, respectively, and the other is the false target whose azimuth angle, elevation angle, and variance are $\theta_{s}\pm \pi$, $\alpha_{s}$, and $\sigma_{s}^{2}/2$, respectively. Consequently, $R_{im}$ contains the actual and false targets. When the output signal intensity is calculated, $R_{is1}$ and $R_{is2}$ should be included simultaneously. The output signal intensity is described as:

(30) $$P_{iso}(\alpha_{s},\theta_{s})=w^{H}(\alpha_{s},\theta_{s})R_{is1}w(\alpha_{s},\theta_{s})-w^{H}(\alpha_{s},\theta_{s})R_{is2}w(\alpha_{s},\theta_{s})\;=\frac{\sigma_{s}^{2}}{2}(1-L_{1}),$$

where $L_{1}$ is defined as loss coefficient:

(31) $$L_{1}=\frac{1}{M^{2}}\sum_{i=1}^{M}e^{-j4\pi r_{\lambda }\sin\alpha_{s}\cos(\beta_{i}-\theta_{s})}\sum_{i=1}^{M}e^{j4\pi r_{\lambda }\sin\alpha_{s}\cos(\beta_{i}-\theta_{s})},$$

where $r_{\lambda }$ is defined as the radius to wavelength ratio. Notably, the loss coefficient is related to $r_{\lambda }$, array element position, and signal direction. When the loss coefficient is equal to 1, $P_{iso}(\alpha_{s},\theta_{s})$ is smallest. However, when the loss coefficient is equal to 0, $P_{iso}(\alpha_{s},\theta_{s})$ is largest, that is $\sigma_{s}^{2}/2$. The quantitative analysis of the loss coefficient is presented in Section 5.

Similarly, According to Equation (23), the imaginary part of the noise covariance matrix is described as:

(32) $$R_{in}={\left(j\sum_{b=1}^{B^{\prime }}P_{klb}\sin(\mu_{klb}\Delta_{kl}(\alpha_{b},\varphi_{b}))\right)}_{M\times M}.$$

Then, the output noise intensity is obtained as:

(33) $$P_{ino}(\alpha ,\theta )=w^{H}(\alpha ,\theta )R_{in}w(\alpha ,\theta ).$$

According to Equations (30) and (33), the output SNR is obtained as:

(34) $$SNR_{io}=\frac{w^{H}(\alpha_{s},\theta_{s})R_{is}w(\alpha_{s},\theta_{s})}{w^{H}(\alpha_{s},\theta_{s})R_{in}w(\alpha_{s},\theta_{s})}=\frac{\sigma_{s}^{2}(1-L_{1})}{2w^{H}(\alpha_{s},\theta_{s})R_{in}w(\alpha_{s},\theta_{s})}.$$

The output noise intensity expressed in Equation (33) reflects the capability to remove symmetrical component. In practice, the output noise intensity is different for various received noises. Thus, the noise removal capacity depends on the characteristic of the noise field. According to Equation (32), the greater the symmetrical component is, the stronger the noise removal capacity is. Although the signal contained in the real part is also lost in Equation (30), the noise loss is always more than the signal loss. As a consequence, the output SNR of the IDAS method is always larger than that of the DAS method by comparing the Equation (34) with the Equation (25). As an example, the noise removal capacity in a spherical isotropic noise field is analyzed in Section 5.

### 4.3. False-Target Elimination Method

The IDAS method contains the actual and false targets. The reconstructed real part of the signal covariance matrix is obtained to eliminate the false target. Then, the reconstructed covariance matrix composed of the imaginary and reconstructed real parts is applied to the DAS method (DAS based on the reconstructed covariance matrix, RCMDAS). Consequently, the false target is eliminated. This section presents two methods: based on the signal variance estimation method (SVE-RCMDAS) and based on the constrained optimization method (CP-RCMDAS).

#### 4.3.1. Based on the Signal Variance Estimation Method (SVE-RCMDAS)

According to Equation (5), the first row of the imaginary part of covariance matrix is denoted as ***Q***, and can be expressed in matrix form, as follows:

(35) $$S(\alpha_{s},\theta_{s})\sigma_{s}^{2}+N_{im}=Q^{T},$$

where $N_{im}^{T}$ is denoted as the first row of $R_{in}$ and $S(\alpha_{s},\theta_{s})$ is denoted as:

(36) $$S(\alpha_{s},\theta_{s})={\left[\begin{array}{cccc} \sin(\Delta_{12}(\alpha_{s},\theta_{s})) & \sin(\Delta_{13}(\alpha_{s},\theta_{s})) & \cdots & \sin(\Delta_{1M}(\alpha_{s},\theta_{s})) \end{array}\right]}^{T}.$$

The estimated azimuth angle of signal ${\hat{\theta }}_{s}$ and elevation angle ${\hat{\alpha }}_{s}$ are obtained from the IDAS method. The actual and false targets are acquired from the IDAS method. The actual target cannot be distinguished; thus, ${\hat{\alpha }}_{s}$ and ${\hat{\theta }}_{s}$ may be the actual target or the false target. By substituting ${\hat{\alpha }}_{s}$ and ${\hat{\theta }}_{s}$ into Equation (35), the linear equation of signal variance estimation $\hat{e}$ is acquired in matrix form:

(37) $$\hat{S}({\hat{\alpha }}_{s},{\hat{\theta }}_{s})\hat{e}+N_{im}=Q^{T}.$$

For notational simplicity, $\hat{S}({\hat{\alpha }}_{s},{\hat{\theta }}_{s})$ is denoted by $\hat{S}$. Then, the minimal norm least square solution of Equation (37) is obtained:

(38) $$\hat{e}={\hat{S}}^{+}(Q^{T}-N_{im})={\hat{S}}^{+}Q^{T}-{\hat{S}}^{+}N_{im}=e^{\prime }-\delta$$

where “+” denotes the generalized inverse, $e^{\prime }={\hat{S}}^{+}Q^{T}$, and $\delta ={\hat{S}}^{+}N_{\mathrm{im}}$.

If ${\hat{\alpha }}_{s}$ and ${\hat{\theta }}_{s}$ are the angles of the actual target, then $\hat{e}$ is positive; if ${\hat{\alpha }}_{s}$ and ${\hat{\theta }}_{s}$ are the angles of the false target, then $\hat{e}$ is negative. As a result, the estimated signal variance is calculated as ${\hat{\sigma }}_{s}^{2}=\left|\hat{e}\right|$.

In practice, $Q^{T}$ and ${\hat{S}}^{+}$ can be obtained. Nevertheless, $N_{im}$ cannot be precisely estimated. Therefore, the signal variance is estimated as:

(39) $${\hat{\sigma }}_{s}^{2}\approx \left|{\hat{S}}^{+}Q^{T}\right|=\left|e^{\prime }\right|=\left|\hat{e}+\delta \right|=\sigma_{s}^{2}+\delta^{\prime }.$$

The estimated signal variance from Equation (39) can be interpreted as the actual signal variance that adds an error $\delta^{\prime }$. The relative error of signal variance estimation is defined as:

(40) $$\Delta_{\delta }=\frac{\left|\delta^{\prime }\right|}{\sigma_{s}^{2}}.$$

In conclusion, the estimated signal variance is affected by the azimuth angle of signal ${\hat{\theta }}_{s}$, elevation angle ${\hat{\alpha }}_{s}$, and relative error $\Delta_{\delta }$. The quantitative analysis of $\Delta_{\delta }$ is presented in Section 5.

According to the estimated signal variance ${\hat{\sigma }}_{s}^{2}$, the reconstructed real part of the signal covariance matrix is obtained as:

(41) $${\hat{R}}_{re}={\hat{\sigma }}_{s}^{2}\rho_{re}=\sigma_{s}^{2}\rho_{re}+\delta^{\prime }\rho_{re}={\hat{R}}_{rs}+{\hat{R}}_{\delta },$$

where ${\hat{R}}_{rs}$ is the real part of the signal covariance matrix, ${\hat{R}}_{\delta }=\delta^{\prime }\rho_{re}$, and $\rho_{re}$ is the normalized ${\hat{R}}_{rs}$, which is denoted as:

(42) $$\rho_{re}={\left(\cos(\Delta_{kl}({\hat{\alpha }}_{s},{\hat{\theta }}_{s}))\right)}_{M\times M}.$$

According to Euler’s formula, ${\hat{R}}_{\delta }$ can be described as the sum of matrices ${\hat{R}}_{\delta 1}$ and ${\hat{R}}_{\delta 2}$, which are described as:

(43) $${\hat{R}}_{\delta 1}={\left(\frac{\delta^{\prime }}{2}e^{j\Delta_{kl}({\hat{\alpha }}_{s},{\hat{\theta }}_{s})}\right)}_{M\times M},\;{\hat{R}}_{\delta 2}={\left(\frac{\delta^{\prime }}{2}e^{-j\Delta_{kl}({\hat{\alpha }}_{s},{\hat{\theta }}_{s})}\right)}_{M\times M}.$$

${\hat{R}}_{\delta 1}$ can be viewed as a signal covariance matrix whose azimuth angle, elevation angle, and variance are ${\hat{\theta }}_{s}$, ${\hat{\alpha }}_{s}$, and $\delta^{\prime }/2$, respectively, which enhances the signal. Meanwhile, ${\hat{R}}_{\delta 2}$ can be viewed as a signal covariance matric whose azimuth angle, elevation angle, and variance are ${\hat{\theta }}_{s}\pm \pi$, ${\hat{\alpha }}_{s}$, and $\delta^{\prime }/2$, respectively, which corresponds to the false target.

According to Equations (27), (32) and (41), the reconstructed covariance matrix is obtained as:

(44) $$\hat{R}={\hat{R}}_{re}+R_{im}={\hat{R}}_{s}+{\hat{R}}_{n},$$

where ${\hat{R}}_{s}={\hat{R}}_{rs}+R_{is}+{\hat{R}}_{\delta 1}$ and ${\hat{R}}_{n}=R_{in}+{\hat{R}}_{\delta 2}$ are denoted as the estimated signal covariance matrix and estimated noise covariance matrix, respectively. Then, the output SNR is obtained as:

(45) $$SNR_{SVE}=\frac{\sigma_{s}^{2}+\frac{\delta^{\prime }}{2}}{w^{H}(\alpha_{s},\theta_{s})R_{in}w(\alpha_{s},\theta_{s})+\frac{\delta^{\prime }}{2}L_{1}}.$$

The output signal intensity of the SVE-RCMDAS method is denoted as $P_{sso}$, which is the sum of $\sigma_{s}^{2}$ and $\frac{\delta^{\prime }}{2}$. Meanwhile, the output noise intensity of the SVE-RCMDAS method is represented as $P_{sno}$. When $\delta^{\prime }$ is large, the numerator and the denominator increase and the undesired false target appears in the direction spectrum. Thus, $\delta^{\prime }$ should be small. The quantitative analysis of the change of the relative error with input SNR is presented in Section 5.

#### 4.3.2. Based on the Constrained Optimization Method (CP-RCMDAS)

Instead of employing the minimal norm least square solution, we introduce another new technique to eliminate the false target based on the constrained optimization method.

An estimated variance of signal ${\hat{\sigma }}_{s}^{2}$ is generated randomly, and $0<{\hat{\sigma }}_{s}^{2}<\lambda_{\max}$, where $\lambda_{\max}$ is the maximum of the diagonal of the covariance matrix. Then, the reconstructed real part of the signal covariance matrix is obtained by using Equation (41) and the reconstructed covariance matrix is acquired by employing Equation (44). The output intensity is achieved:

(46) $$P({\hat{\sigma }}_{s}^{2},\alpha ,\theta )=w^{H}(\alpha ,\theta )\hat{R}w(\alpha ,\theta ).$$

According to Equation (44), the output in the direction of the actual target is calculated as:

(47) $$P_{rs}=w^{H}(\alpha_{s},\theta_{s}){\hat{R}}_{s}w(\alpha_{s},\theta_{s})=\sigma_{s}^{2}+\frac{\delta^{\prime }}{2},$$

and the output in the direction of the false target is obtained:

(48) $$P_{ri}=w^{H}(\alpha_{s},\theta_{s}\pm \pi ){\hat{R}}_{\delta 2}w(\alpha_{s},\theta_{s}\pm \pi )=\frac{\delta^{\prime }}{2}.$$

Comparing Equation (47) with Equation (48), $P_{rs}>P_{ri}$ can be observed. As a result, the output in the direction of the actual target is maximum. Thus, the constrained optimization problem for obtaining ${\hat{\sigma }}_{s}^{2}$ can be formulated as follows:

(49) $$\underset{{\hat{\sigma }}_{s}^{2}}{\min}\;\underset{\begin{array}{l} \left|\theta -{\hat{\theta }}_{s}\right|>\Delta_{\theta }\\ \left|\alpha -{\hat{\alpha }}_{s}\right|>\Delta_{\alpha } \end{array}}{\max}\left|P({\hat{\sigma }}_{s}^{2},\alpha ,\theta )\right|,\;s.t.\;0<{\hat{\sigma }}_{s}^{2}<\lambda_{\max},$$

where $\Delta_{\theta }$ and $\Delta_{\alpha }$ denote the main-lobe width. The constrained optimization problem is solved by using the particle swarm algorithm. The output SNR denoted as $SNR_{CP}$ of the CP-RCMDAS method is obtained by using Equation (45), and the output signal intensity is denoted as $P_{cso}$. The advantage of this method is that it avoids the estimation of the signal variance; thus, its performance is stable. However, the disadvantage of this method is its slow computing speed because of the optimization process.

## 5. Simulation Experiment

For an *M*-element UCA, the eigen wavelength $\lambda_{t}$ is generally defined as:

(50) $$\lambda_{t}=2d_{cr}\Rightarrow r_{t}=\frac{M}{4\pi },$$

where $d_{cr}$ denotes the arc length between two adjacent elements and $r_{t}$ denotes the eigen radius-to-wavelength ratio that corresponds to $\lambda_{t}$. 

**Figure 2:**
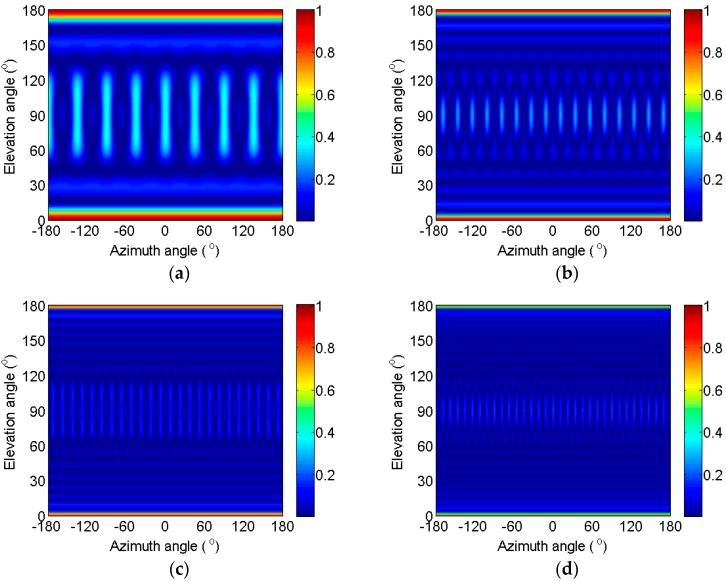
Change of the loss coefficient with the azimuth and elevation angles. (**a**) *M* = 8, $r_{\lambda }=2/\pi$; (**b**) *M* = 16, $r_{\lambda }=4/\pi$; (**c**) *M* = 24, $r_{\lambda }=6/\pi$; (**d**) *M* = 32, $r_{\lambda }=8/\pi$.

When M=8,16,24,32 and the radius is equal to 2 m, $r_{t}=2/\pi ,4/\pi ,6/\pi ,8/\pi$, respectively. The change of the loss coefficient with the azimuth and elevation angles is shown in Figure 2. The estimated capacity of the azimuth angle worsens and the loss coefficient is approximately equal to 1 when the elevation angle is in the area near 0° or 180°. However, this case is less common and make no sense. Apart from this area, the loss coefficient clearly reaches its second maximum when the elevation angle is near 90°. The elevation angle is selected as 90° in the following context of this section because the worst performance should be considered.

Next, the change of the loss coefficient with the azimuth angle when the elevation angle is selected as 90° is discussed in this paragraph. According to the characteristics of the UCA and Equation (31), the loss coefficient can vary regularly with the change of the azimuth angle. *M* peaks and *M* valleys exist for an *M*-element UCA. When the target is located in the direction that corresponds to the valley, the loss coefficient is small, and the output signal intensity of the IDAS method is approximately equal to $\sigma_{s}^{2}/2$, according to Equation (30). The peak value and width decrease with the increase of the number of elements. The peak values are 0.38, 0.28, 0.13, and 0.11 in Figure 2a–d, the corresponding signal losses are 5.09, 4.44, 3.62, and 3.52 dB, respectively, according to Equation (30).

**Figure 3:**
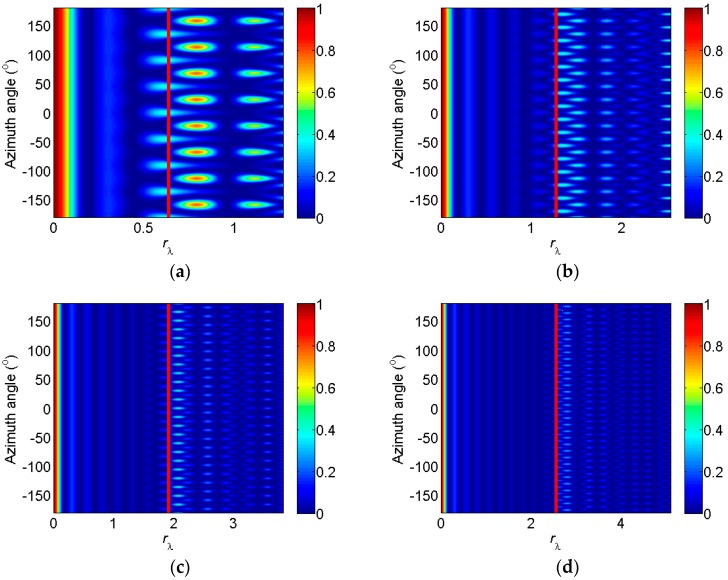
Change of the loss coefficient with $r_{\lambda }$ and azimuth angle. (**a**) *M* = 8; (**b**) *M* = 16; (**c**) *M* = 24; (**d**) *M* = 32.

Then, the frequency characteristics of the loss coefficient are analyzed. The change of the loss coefficient with $r_{\lambda }$ and the azimuth angle is shown in Figure 3, with the red lines representing $r_{t}$. From this figure, the loss coefficient is large when $r_{\lambda }$ is small, because the small $r_{\lambda }$ leads to the small exponent in Equation (31). Consequently, the IDAS method cannot be used and make no sense. Apart from the area of small $r_{\lambda }$, the second maximum decreases with the increase of the number of elements, and the corresponding $r_{\lambda }$ is larger than $r_{t}$. Between the maximum and second maximum, the area where the loss coefficient is small, is called the feasible area of the IDAS method. The comparison of these four figures shows that the width of the feasible area increases with the increase of the number of elements.

According to Figure 2 and Figure 3, the signal loss can be predicated by selecting the number of elements, radius, and working frequency. Next, the noise removal capacity is analyzed, furthermore, the output SNR is obtained.

**Figure 4:**
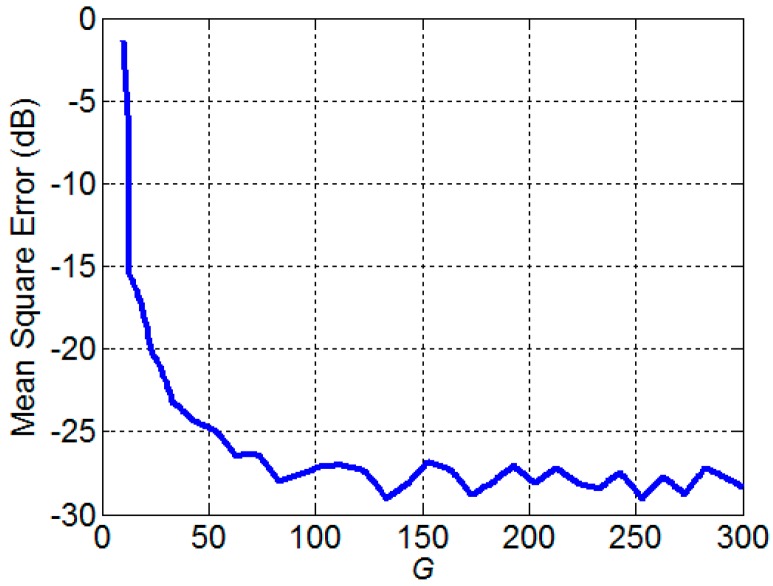
The normalized mean square error between the spatial coherence calculated from the noise model and the theoretical spatial coherence.

Firstly, the error between the noise modeled by a number of uncorrelated noise sources and the actual noise should be discussed. In this paper, the isotropic noise field is taken as an example. The well-known theoretical spatial coherence function for spherically isotropic noise and omnidirectional sensors is described as:

(51) $$r(d/\lambda )=\frac{\sin(2\pi d/\lambda )}{2\pi d/\lambda },$$

where *d* is the distance of two sensors, and λ is the wavelength. The error between the spatial coherence calculated from the noise model and the theoretical spatial coherence is defined by the normalized mean square error (MSE) between these two values [25], that is:

(52) $$\mathrm{MSE}(G)=\frac{\sum_{k=0}^{K/2}{\left(r(f_{k})-\hat{r}(f_{k},G)\right)}^{2}}{\sum_{k=0}^{K/2}r^{2}(f_{k})},$$

where *K* denotes the number of discrete frequencies and $\hat{r}(f_{k},G)$ denotes the estimated spatial coherence obtained by using *G* noise sources. The result is shown in Figure 4. For a large *G*, the MSE asymptotically reaches a certain level. In case the number of noise sources is larger than 100, the theoretical spatial coherence is well approximated. *G* is selected as 300 in the subsequent simulation experiments.

**Figure 5:**
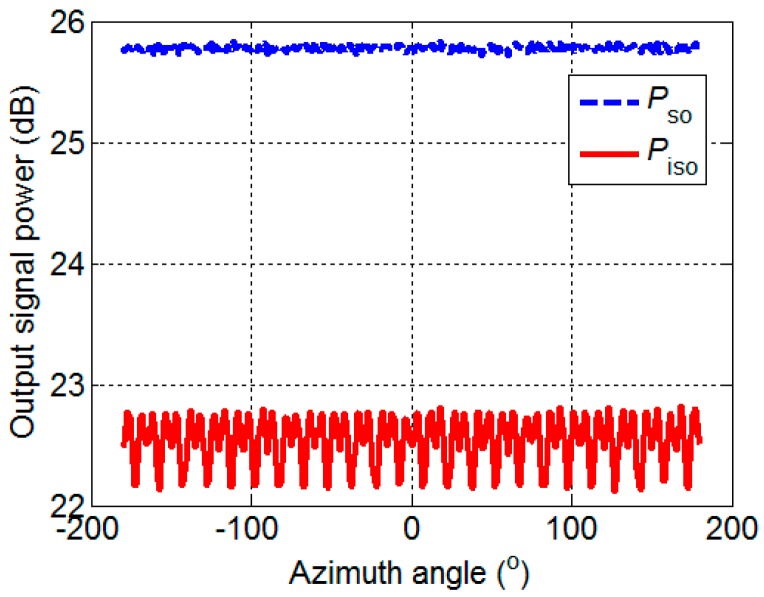
The output signal.

Then, a 24-element UCA is given, the radius is 2 m, the center frequency of the narrowband signal and noise is 2000 Hz, the bandwidth of signal is 50 Hz, the bandwidth of noise is 100 Hz, and the sampling frequency is 16 kHz. The SNR is defined as the variance ratio. The input SNR is assumed to be 0 dB, and the changes of the $P_{so}$ and $P_{iso}$ with the azimuth angle are shown in Figure 5. The $P_{iso}$ varies regularly with the change of the azimuth angle, which corresponds to the same law of loss coefficient. The comparison of the $P_{so}$ with $P_{iso}$ shows that the maximum signal loss is 3.61 dB and the minimum signal loss is 3.03 dB.

**Figure 6:**
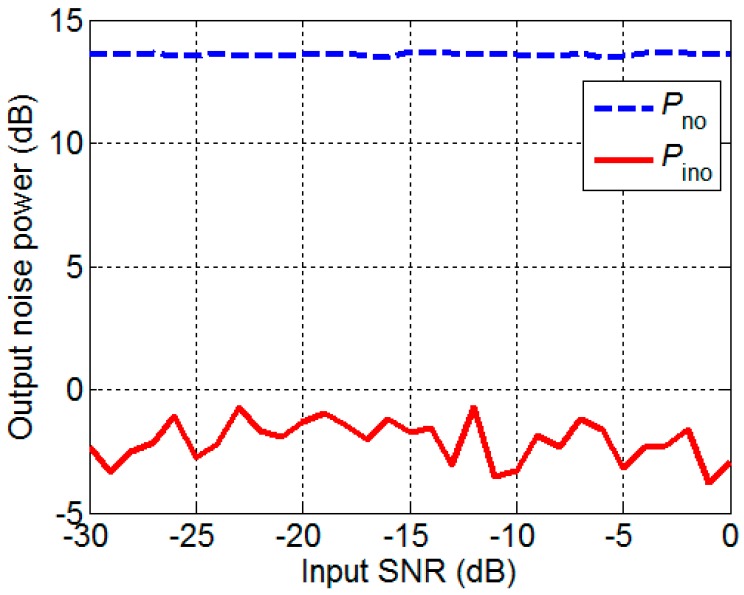
The output noise.

The azimuth angle is assumed to be 80°, and the changes of the $P_{no}$ and $P_{ino}$ with the input SNR are shown in Figure 6. Both $P_{no}$ and $P_{ino}$ do not vary with the input SNR. The $P_{ino}$ is less than $P_{no}$ by approximately 16 dB, which is a significant value. The isotropic noise field is simulated in this paper, such that the symmetrical component is large according to the definition of the symmetrical component. According to the principle of noise covariance matrix decomposition, the greater the symmetrical component is, the stronger the noise removal capacity is. Therefore, 16 dB is credible. According to Figure 5 and Figure 6, the output SNR is improved by using IDAS method.

**Figure 7:**
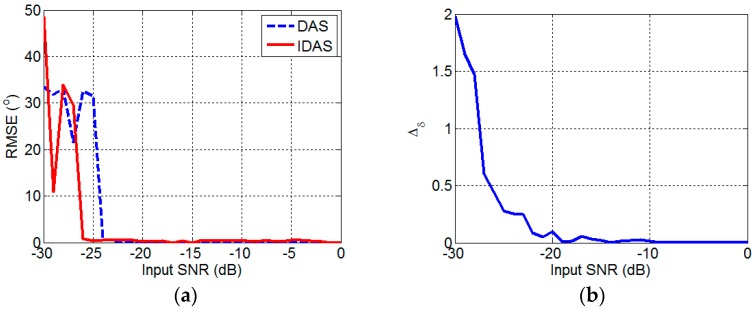
Errors. (**a**) ${\hat{\theta }}_{s}$ and ${\hat{\alpha }}_{s}$ RMSEs; (**b**) $\Delta_{\delta }$.

Then, the $\Delta_{\delta }$, ${\hat{\theta }}_{s}$, and ${\hat{\alpha }}_{s}$ should be analyzed because the performance of the RCMDAS method is based on these factors. Figure 7a presents the change of the sum of the ${\hat{\theta }}_{s}$ and ${\hat{\alpha }}_{s}$ RMSEs with the input SNR. When the input SNR is large, the RMSEs of the DAS and IDAS methods are small. The RMSE of the DAS method increases, whereas that of the IDAS method varies only slightly with the decrease of the input SNR. As the input SNR continues to decrease, the RMSEs of both methods are large. This result reveals that the IDAS method provides much better performance in direction of arrival estimation than DAS method. Figure 7b presents the change of $\Delta_{\delta }$ with the input SNR. $\Delta_{\delta }$ decreases with the increase of the input SNR. This result reveals that the performance of SVE-RCMDAS method becomes better. According to Equation (37), the ${\hat{\theta }}_{s}$ and ${\hat{\alpha }}_{s}$ RMSEs affect the $\Delta_{\delta }$. However, according to Equation (41), the $\Delta_{\delta }$ affects the reconstructed real part matrix, which will affect the output intensity of the SVE-RCMDAS. According to Equation (49), the ${\hat{\theta }}_{s}$ and ${\hat{\alpha }}_{s}$ RMSEs influence the result of the CP-RCMDAS method. As a result, the small ${\hat{\theta }}_{s}$ and ${\hat{\alpha }}_{s}$ RMSEs and the small $\Delta_{\delta }$ provide the basis for the application of the RCMDAS method.

**Figure 8:**
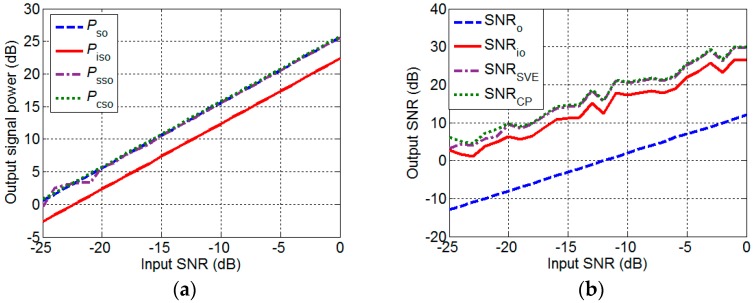
Comparison of the DAS, IDAS, SVE-RCMDAS, and CP-RCMDAS methods. (**a**) Output signal intensity; (**b**) Output SNR.

Figure 8a presents the changes of the $P_{so}$, $P_{iso}$, $P_{sso}$, and $P_{cso}$ with the input SNR. The $P_{sso}$ and $P_{cso}$ are approximately equal to that of the DAS method, and the output signal intensity of the IDAS method is smaller by 3.2 dB than those of the other three methods. When the input SNR is low, the output of the SVE-RCMDAS method appears perturbations because the low input SNR leads to significant error of the estimated signal variance. This case does not exist in the curve of the CP-RCMDAS method. According to Figure 6 and Figure 8a, the changes of the output SNRs of the DAS ($SNR_{o}$), IDAS ($SNR_{io}$), SVE-RCMDAS ($SNR_{SVE}$), and CP-RCMDAS ($SNR_{CP}$) methods with input SNR can be obtained, as shown in Figure 8b. The output SNR of the DAS method is the smallest, that of the IDAS method comes second, and those of the RCMDAS methods are the largest.

## 6. Experimental Results

An experimental 16-hydrophone UCA is used in the South China Sea with two sea conditions, in which the radius is 1.5 m and the sampling frequency is 25 kHz. The sea depth of the experimental point is 2000 m, and the UCA is deployed in a depth of 300 m. The launch device is a fish lip transducer that is deployed in a depth of 50 m. The horizontal distance between the launch devices with the UCA is 600 m. 

**Figure 9:**
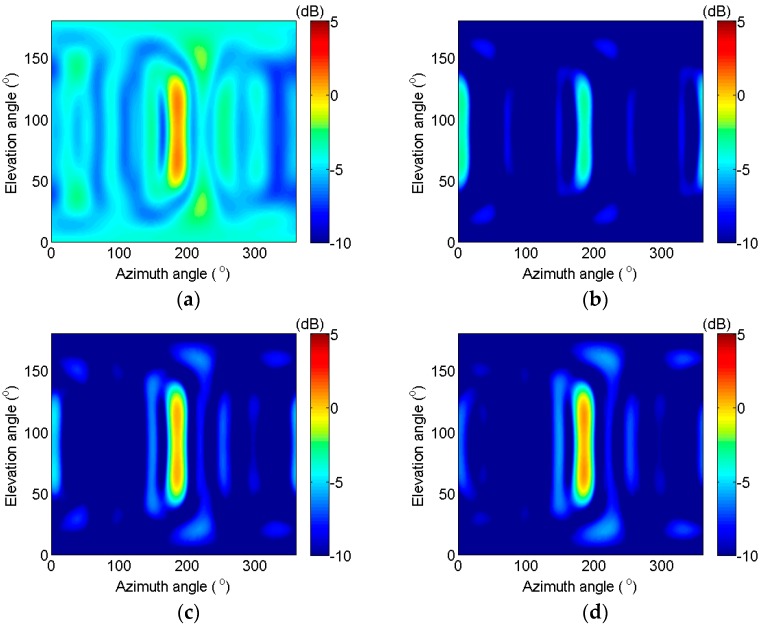
Direction Spectra of the four methods. (**a**) DAS method; (**b**) IDAS method; (**c**) SVE-RCMDA method; (**d**) CP-RCMDAS method.

The emission signal is a chirp signal with a center frequency of 950 Hz and bandwidth of 500 Hz, the time length of the chirp signal is 1.5 s and the cycle is 2 s. Figure 9 shows the direction spectra of the DAS, IDAS, SVE-RCMDAS, and CP-RCMDAS methods when the data with 2 s is used. We can find that there exist the up-down ambiguity, because the directive map of the DAS method for the UCA exists the up-down ambiguity. The output of the DAS method is presented in Figure 9a and that of the IDAS method is exhibited in Figure 9b. The elevation angles of the direct wave and the surface-bounce wave are close to each other and the multi-target resolution of the DAS method is restricted by Rayleigh limit, therefore, we cannot distinguish the direct wave and the surface-bounce wave from the Figure 9a. When (a) is compared with (b), the output of the DAS method in the direction of the actual target is larger than that of the IDAS method. Furthermore, the output of the IDAS method in the direction of the false target is approximately equal to that of the actual target, and the difference between the azimuth angles of the actual target and the false target is approximately equal to 180°. As a result, the azimuth angle of the actual target is difficult to distinguish. Apart from the directions of the actual and false targets, the outputs in the other directions are considered the approximation of the output noise intensity. Notably, the approximate output noise intensity of the IDAS method is less than that of the DAS method by more than 6.4 dB. The output of the SVE-RCMDAS method is presented in Figure 9c. When (c) is compared with (b), the false target is almost eliminated and the output in the direction of the actual target increases. When (c) is compared with (a), the approximate output noise intensity of the SVE-RCMDAS method is less than that of the DAS method, and the output of the RCMDAS method in the direction of the actual target is approximately equal to that of the DAS method, which reveals that the noise is removed and the signal is distortionless. The output of the CP-RCMDAS method is presented in Figure 9d. When (d) is compared with (c), the CP-RCMDAS method exhibits all advantages of the SVE-RCMDAS method. Moreover, the false target is eliminated more thoroughly by using the CP-RCMDAS method.

The output SNR is calculated by using the approximation method. First, the sums of the output noise intensity and output signal intensity are obtained from the data with signal. Second, with the use of the weighted vector of the previous step, the output noises of the DAS and IDAS methods are calculated from the data without signal, and the output noises of the two RCMDAS methods are assumed to be the same as those of the IDAS method. Finally, the output SNR is listed in Table 1. 

From Table 1, the following preliminary conclusions are drawn: (1) The output SNR of the IDAS method is larger than that of the DAS method by 2.15 dB, which is a comprehensive result of the signal loss and noise removal. (2) The output SNR of the IDAS method is smaller than that of the SVE-RCMDAS and CP-RCMDAS methods by 3.514 and 3.781 dB, respectively, which is the result of the addition of the reconstructed real part of signal covariance matrix.

**Figure 10:**
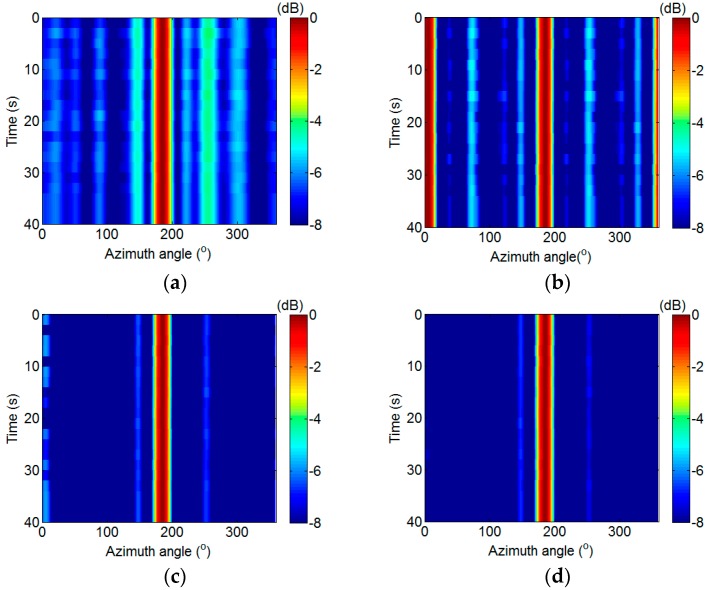
BTR figures. (**a**) DAS method; (**b**) IDAS method; (**c**) SVE-RCMDAS method; (**d**) CP-RCMDAS method.

The data with 40 s is used in the DAS, IDAS, SVE-RCMDAS, and CP-RCMDAS methods. The corresponding bearing time record (BTR) figures, when the elevation angle is equal to 68° according to Figure 9, are shown in Figure 10a–d.

The normalized side lobe level of BTR (BSLL) is defined as:

(53) $$BSLL=10\mathrm{lg}\left(\frac{1}{N_{T}}\frac{1}{N_{\theta }}\sum_{i=1}^{N_{T}}\sum_{j=1}^{N_{\theta }}P(t_{i},\theta_{j})\right),$$

where $t_{i}$ and $\theta_{j}$ denote the discrete time and azimuth angle, respectively; $N_{T}$ and $N_{\theta }$ represent the sampling number of time and azimuth angle, respectively; and $P(t_{i},\theta_{j})$ denotes the normalized output. Figure 10a presents the BTR figure of the DAS method. A target whose azimuth angle is 185° and BSLL is −5.02 dB can be detected. Figure 10b presents the BTR figure of the IDAS method. The false target appears and the BSLL, which is equal to −7.84 dB, is smaller than that of the DAS method. Figure 10c,d present the BTR figures of the SVE-RCMDAS and CP-RCMDAS methods, respectively. The false target is eliminated, and the BSLL of the CP-RCMDAS method, which is equal to −10.18 dB, is smaller than that of the SVE-RCMDAS method, which is equal to −9.31 dB.

In summary, the SVE-RCMDAS method performs worse than the CP-RCMDAS method in terms of the output SNR and the BSLL. However, the SVE-RCMDAS method performs better than the CP-RCMDAS method in terms of computing time, as shown in Table 2.

## 7. Conclusions

The array signal processing method is applied under underwater ambient noise. The received noises of two arbitrary array elements are correlated, as a consequence, the noise removal capacity may be deteriorated. A noise removal method is proposed to solve the previously presented problem. The preliminary conclusions are summarized as follows:

(1) The noise covariance matrix can be decomposed into symmetrical and asymmetrical components, and the symmetrical component can affect only the real part of the covariance matrix. The real part of the covariance matrix is eliminated, and the imaginary part is used in array signal processing. Thus, the symmetrical noise is removed.
(2) For a UCA, the signal loss by using the IDAS method is analyzed, which provides a basis for the UCA design and the working frequency selection.
(3) The IDAS method performs better than the DAS method, the noise is removed, and the output SNR increases.
(4) The IDAS method produces a false target. The difference between the azimuth angles of the actual and false targets is equal to 180°; thus, the azimuth angle of the actual target cannot be distinguished.
(5) Two methods for the reconstruction of the signal covariance matrix are presented to eliminate the false target: SVE-RCMDAS and CP-RCMDAS. The performance of SVE-RCMDAS method is bad when the input SNR is low. By contrast, the performance of CP-RCMDAS method is better than that of the SVE-RCMDAS method.
(6) The theoretical analysis and experimental results show that the IDAS method is easy to implement and improves the noise removal capacity of array signal processing.
(7) The experimental results show that the output SNR of the IDAS method increases by 2.15 dB, and those of the SVE-RCMDAS and CP-RCMDAS methods increase by 5.664 and 5.931 dB, respectively.

## Figures and Tables

**Table 1:** Output SNR.

Method	Output SNR (dB)
DAS	2.837
IDAS	4.987
SVE-RCMDAS	8.501
CP-RCMDAS	8.768

**Table 2:** Computing time (unit: seconds).

Method	Computing Time
SVE-RCMDAS	13.70
CP-RCMDAS	357.70
